# Antibiotic-Loaded PMMA Beads for Recurrent Sternocutaneous Fistula: Expanding the Surgical Armamentarium in Post-Sternotomy Osteomyelitis: Case Report and Literature Review

**DOI:** 10.3390/life15101547

**Published:** 2025-10-02

**Authors:** Mircea Robu, Irina Maria Margarint, Andrei Draganita, Miruna Guzu, Vlad Anton Iliescu

**Affiliations:** 1 Faculty of Medicine, Carol Davila University of Medicine and Pharmacy, 050474 Bucharest, Romania; mircea.robu@drd.umfcd.ro (M.R.); vlad.iliescu@umfcd.ro (V.A.I.); 2 Department of Orthopedics, Giurgiu Emergency Hospital, 080302 Giurgiu, Romania; andrei.draganita@gmail.com; 3 Department of Cardiac Surgery, “Prof. Dr. C. C. Iliescu” Emergency Institute for Cardiovascular Diseases, 022328 Bucharest, Romania; guzu.miruna@gmail.com

**Keywords:** sternocutaneous fistula, chronic sternal osteomyelitis, post-sternotomy infection, antibiotic-loaded PMMA beads, cardiac surgery complication

## Abstract

Background: Late sternocutaneous fistulas (SCFs), secondary to chronic sternal osteomyelitis, are uncommon sequelae of median sternotomy and present significant therapeutic challenges. They are frequently linked to low-virulence microorganisms forming biofilms on retained foreign materials. While antibiotic-impregnated polymethylmethacrylate (PMMA) beads are established in managing chronic osteomyelitis in other anatomical locations, reports describing their use for post-sternotomy SCFs are limited to two early postoperative cases. Case Presentation: We describe a 62-year-old man with a history of triple-vessel coronary artery disease who underwent coronary artery bypass grafting via median sternotomy. Two months postoperatively, he developed an SCF in the upper sternum, initially treated with wire removal, negative pressure wound therapy, and intravenous vancomycin. Recurrence occurred one month later without systemic signs of infection. Imaging revealed inflammatory changes at the level of the manubriosternal junction. Definitive surgery included extensive sternal and costosternal debridement, bilateral anterior arthrolysis of the second ribs, and pulse lavage with 10 L of Microdacyn. The remaining defect was filled with vancomycin- and gentamicin-loaded PMMA beads. The patient had an uneventful recovery with no recurrence at six months. Conclusions: This case suggests that local antibiotic delivery via PMMA beads can be a valuable adjunct in the surgical management of recurrent, late-presenting SCFs after cardiac surgery.

## 1. Introduction

Sternal chronic osteomyelitis with secondary sternocutaneous fistula (SCF) is an uncommon but serious late complication following median sternotomy [1]. They can develop months or even years after surgery [1,2], often without prior surgical site infection history, and have a reported incidence ranging from 0.23% to 0.25% at one year in large cohort studies [1,3]. SCFs after cardiac surgery have a high burden on the medical system because they usually require prolonged antibiotherapy and multiple surgical interventions and significantly decrease the quality of life and survival of these patients [4].

The pathogenesis most frequently involves coagulase-negative staphylococci (CNS) [1,3], which form biofilms on foreign materials such as sternal wires, epicardial pacing leads, or bone wax [5,6]. These biofilms promote persistent, low-grade infection and antimicrobial tolerance. Recognized risk factors include prior SSI, renal dysfunction, smoking, and intraoperative use of bone wax [1,3].

Treatment is not standardized and ranges from prolonged conservative antibiotic regimens in selected comorbid patients [7] to radical surgical debridement with removal of infected bone and foreign material. Local antibiotic carriers, such as PMMA beads impregnated with antibiotics, have shown high eradication rates in chronic osteomyelitis [8,9,10,11]. However, to our knowledge, their use for post-sternotomy sternal osteomyelitis has been reported in only two previous cases in the literature, both involving early deep sternal wound infections [12].

Here, we present the case of recurrent late SCF after coronary artery bypass grafting surgery, successfully managed with extensive surgical debridement and vancomycin- and gentamicin-loaded PMMA beads, combined with targeted systemic antimicrobial therapy.

## 2. Case Report

A 62-year-old patient with a prolonged history of angina, with a past medical history of acute coronary syndrome, hypertension, hyperlipidemia, and active smoking, was admitted to our center. The patient was diagnosed with severe tricoronary vessel disease with indication for myocardial revascularization. He had a good biventricular function with no significant valvulopathies. Miocardial revascularization using two saphenous vein grafts and the left mammary artery was performed via a standard median sternotomy, without any perioperative complications. The patient was discharged on the 7th day after surgery.

**Figure 1:**
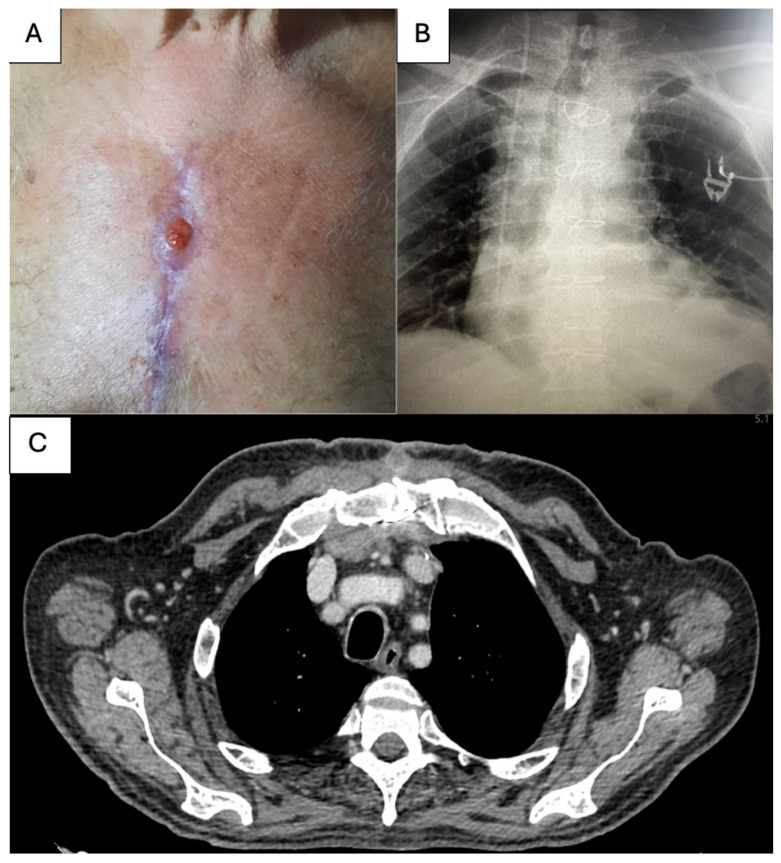
(**A**) Clinical presentation of SCF; (**B**) Chest XR at presentation; (**C**) Chest CT aspect at presentation.

After two months, the patient returned to our clinic with an SCF in the superior half of the sternal wound with minimal purulent discharge (Figure 1A). Clinical examination was within normal limits, and the patient did not report fever at home. The patient did not have sternal instability. Laboratory findings revealed no inflammatory syndrome, and the culture harvested from the SCF was negative. A chest RX (Figure 1B) and a chest CT (Figure 1C) were obtained. On the anteroposterior chest radiograph, the sternal cerclage wires from the previous surgical intervention are visualized and appear intact. No sternal fracture lines are identified. At the level of the sternal manubrium and body, irregular cortical margins and heterogeneous density are noted, findings that may reflect postoperative remodeling. The adjacent costosternal articulations, particularly on the right side, demonstrate altered contour and sclerosis, features suggestive of postsurgical changes with possible inflammatory or infectious sequelae. On chest CT, a small dense collection located posterior to the sternal manubrium, with maximum axial dimensions of approximately 50 × 20 mm (measured at the level of the horizontal segment of the left brachiocephalic vein), extending cranio-caudally over a length of ~70 mm, was described. The collection extends anteriorly to the presternal region, reaching the skin surface through the sternotomy tract at the level of the second sternocostal joint.

The patient was initiated on intravenous vancomycin therapy. The sternal steel wire corresponding to the SCF was surgically removed, followed by the application of a negative pressure wound therapy (NPWT) dressing. On postoperative day five, a repeat culture from the sternal wound was obtained, yielding negative results. Given the satisfactory macroscopic wound appearance and the absence of clinical or laboratory evidence of inflammation, definitive wound closure was performed. The patient was discharged in stable condition two days later.

**Figure 2:**
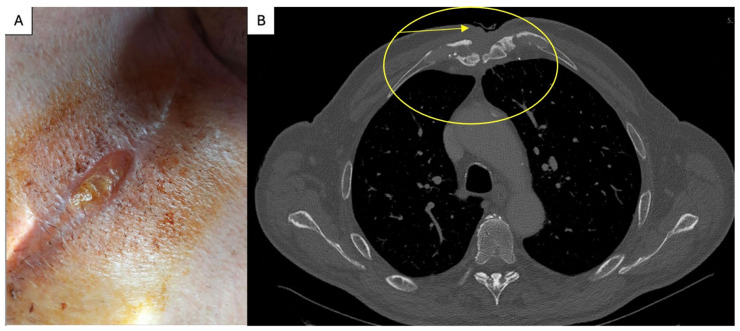
(**A**) SCF at second presentation; (**B**) Axial chest CT showing manubriosternal irregularity with a hypodense tract extending to the skin, consistent with fistulous communication and low-grade inflammation (arrow); the circle represents the area at the anterior chest wall were the SCF is located.

After one month, the patient presented to our institution with a recurrent SCF in the upper half of the sternal wound (Figure 2A). As last time, the clinical condition of the patient was normal. The patient’s hypertension was medically controlled with a combination of a beta-blocker, a diuretic, and an angiotensin-converting enzyme inhibitor. At presentation, the patient had a mean arterial pressure of 55 mmHg, was in sinus rhythm with a heart rate of 70 bpm, and pulmonary examination revealed no pathological findings. Oxygen saturation was 99% on room air. The blood test showed no inflammatory syndrome (C reactive protein = 0.65 mg/L, procalcitonin = 0.02 ng/mL, WBC = 5.9 * 10^3^/μL), there was no sternal instability, and the cultures taken from the level of the SCF were negative. On axial chest CT at the level of the manubrium (Figure 2B), postoperative sternal changes were observed, including irregular and interrupted cortical margins, heterogeneous trabecular density, and mild manubriosternal diastasis. The adjacent second costosternal joints—predominantly on the right—demonstrated contour irregularity and sclerosis, consistent with chronic postoperative remodeling. Within the presternal and parasternal soft tissues, a hypodense, tract-like structure extended toward the skin surface, corresponding to the clinically evident fistulous opening. Surrounding soft tissue changes included mild fat stranding and subtle thickening, indicative of low-grade inflammatory activity. No well-defined fluid collections were identified. Overall, the constellation of findings was most consistent with chronic sternal osteomyelitis, with possible persistent low-grade infection.

**Figure 3:**
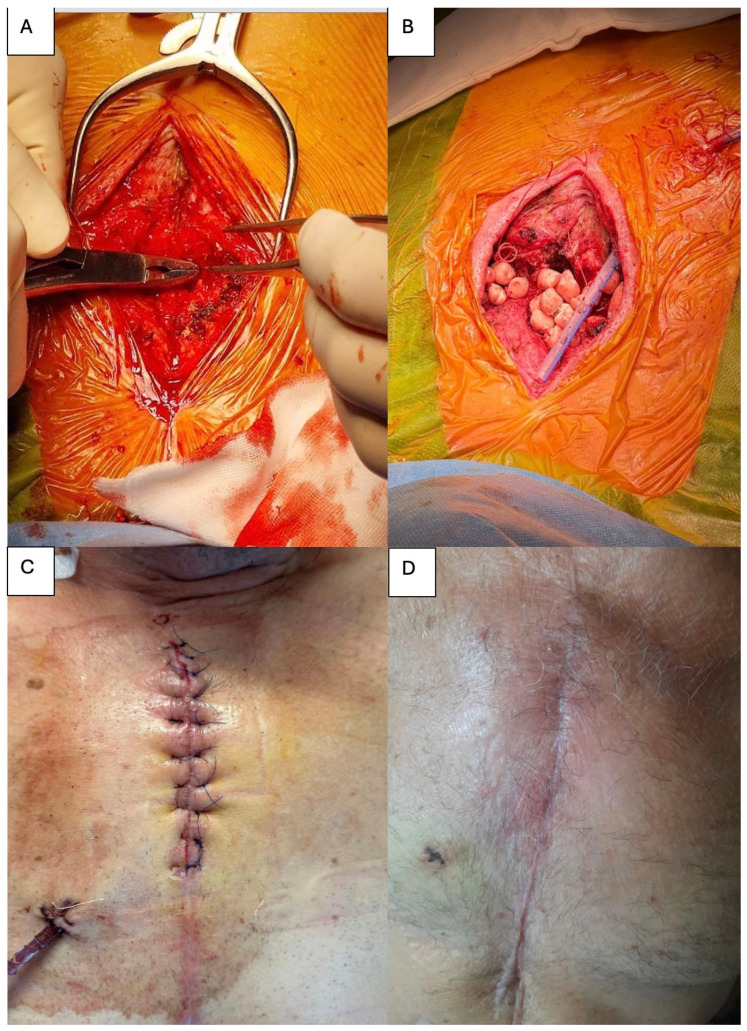
(**A**) Intraoperative view demonstrating extensive debridement of the sternum. (**B**) Placement of antibiotic impregnated PMMA beads within the residual sternal defect. (**C**) Appearance of the sternal wound at the completion of the surgical procedure. (**D**) Clinical aspect of the wound at one-month follow-up.

Following consultation with an infectious disease specialist, intravenous vancomycin and gentamicin therapy was initiated. After a thorough review of the CT findings, the patient was taken to the operating room where under general anesthesia, extensive debridement of the superior portion of the sternal body and the inferior aspect of the manubrium was performed using a rongeur. Bilateral anterior costosternal arthrolysis was subsequently carried out at the level of the second ribs (Figure 3A). Pulse-lavage irrigation of the surgical site was then performed with 10 L of Microdacyn (hypochlorous acid and sodium hypochlorite), a neutral-pH, super-oxidized solution with broad-spectrum antimicrobial activity and established safety for viable tissues. The residual osseous defect was filled with PMMA beads impregnated with vancomycin and gentamicin (Figure 3B). Also, a small drain was placed. The subcutaneous tissue was approximated with interrupted absorbable sutures, and the skin was closed with interrupted 3-0 polypropylene sutures (Figure 3C).

Patient evolution was without any perioperative complications. Cultures taken from deep sternal tissue identified *Staphylococcus warneri*. The drain was removed after two days, and intravenous therapy was continued for a total of one month. After discharge, the patient received an oral antibiotic regimen consisting of trimethoprim-sulfamethoxazole and rifampicin for another 4 weeks. At 1-month (Figure 3D) and 6-month follow-up, no signs of fistula were observed.

## 3. Discussion

Sternal chronic osteomyelitis causing SCFs following median sternotomy represents a rare and serious complication. Late SCFs were first reported in 1983 by Herrera et al. [1] and have a great impact on the patient and health care system because they involve multiple hospitalizations, prolonged antibiotic treatment, and repeated surgical interventions [13,14,15]. Furthermore, Steingrímsson et al. report a significant five-year survival difference between patients with and without SCFs (58% 1% vs. 85% 4%, *p* = 0.003), even after treatment [4].

Superficial and deep SSI are considered early complications according to the Centers for Disease Control if they occur within 30 days of surgery and are the most frequent [16]. The definition of late SSIs is less well defined and can occur months, even years, after median sternotomy. There is little data regarding the subject of late SCF. Most of the data in the literature deals with the subject of early SSI and briefly mentions this complication, usually as case reports [2]. Furthermore, various terms such as draining or chronic sinus, delayed septic costochondritis, recurrent sternal infection, non-healing sternum, sternal osteomyelitis, chondritis, and subcutaneous abscesses are used to describe late SCFs, with significant clinical and pathological overlap among them [15,17,18,19]. The actual definition reported in the literature is that SCFs are draining sinus tracts from the sternum to the skin, treated surgically after discharge from the hospital with an apparent heald sternotomy [20]. Another description is a chronic, low-virulence infection in the sternum, with draining sinus tracts weeks, months, and even years after initial discharge [1], while Tocco et al. refer to this complication as chronic sternal osteomyelitis [7]. SCFs can be classified as primary, occurring without a previous history of SSI, or as secondary to such infection [1,17]. Primary late SCFs are very rare, with a reported 0.23% incidence at one year [1] and have a high risk of occurrence [2]. Similarly, Steingrímsson et al. report an incidence of 0.25% in a nationwide cohort [3], while Tacco et al. communicate 70 patients with late SCFs over a period of 4 years [7]. The real incidence has to be determined, considering that late SCFs can appear even years after median sternotomy [21].

### 3.1. Etiology of CFS

Literature on late primary CFS etiology is scarce, and generally, the focus is on organisms involved in the pathogenesis of SSI. *Staphylococcus epidermidis* is the most frequent, followed by *Staphylococcus aureus* and Gram-negative bacteria. Less common, streptococci, anaerobic bacteria, fungi (*Candida albicans*, *Aspergillus*), and *Mycobacterium tuberculosis* are reported [10,11].

Steingrímsson et al. report that Coagulase-negative staphylococci (CNS) are reported as the most frequent pathogens involved in late CFS occurrence [1] in 32 patients. In another study analysis a nationwide cohort, Steingrímsson et al. report 6 cases, with *Staphylococcus aureus* and/or coagulase-negative staphylococci identified as pathogens in 5 cases and *Candida albicans* in 1 [3]. Tabaković et al. report 3 cases of late SCFs CFS, one with *Pseudomonas aeruginosa* and *Staphylococcus aureus*, and the other two with *Staphylococcus aureus* [2]. Tacco et al. report in 70 patients with chronic osteomyelitis after median sternotomy *Staphylococcus* species in 67% of cases, *Corynebacterium* species in 10% of cases, always associated with the presence of CNS species, Gram-negative species in 21% of cases, fungi in 6% of patients, one patient had a mycobacteria other than tuberculosis infection and negative cultures in 10% of patients [7].

SCFs have been associated with infections involving foreign body implants such as steel wires, epicardial pacemaker leads, or bone wax. In cases involving CNS, the microorganism adheres to the foreign material, facilitates biofilm formation, and results in a low-virulence infection with a typically slow and progressive onset. Typically, the host immune system can eradicate the infection, but in a patient with a compromised immune system, the infection can persist [5,6].

### 3.2. Risk Factors

The majority of patients who develop primary SCFs have a compromised immune system with risk factors for poor wound healing [3]. These risk factors are well described in the literature [4,22]. Preoperative factors like diabetes mellitus, obesity, peripheral arterial disease, smoking, low left ventricular ejection fraction, chronic renal failure, chronic pulmonary disease, intraoperative factors like bilateral use of internal mammary arteries, and postoperative factors like longer ventilator support, use of β-adrenergic drugs, are all involved in the pathogenesis of SSI. Few studies report risk factors for late SCFs. Steingrímsson et al. report that previous SSI, renal failure, smoking, and use of bone wax are major risk factors after analyzing 12,297 patients with median sternotomy [1]. He comments that the majority of patients with late SCFs do not have a history of SSI, and no difference in time to surgical intervention for SCFs was observed between patients with and without previous SSI, suggesting the susceptibility of these patients for poor wound healing. Traditional risk factors for SSI, like diabetes, sex, use of bilateral mammary arteries, and chronic obstructive lung disease, were not associated with late SCFs in the study.

Other risk factors identified were advanced age reported by Peivandi et al. [19] and redo surgery communicated by Jones et al. [23]. Neither study analyzed risk factors specific to late SCFs but rather included this complication in the deep SSI group.

### 3.3. Treatment

Management of SCFs is not standardized, and studies report different strategies, varying from conservative antibiotic treatment to invasive surgery.

Conservative treatment without surgical intervention is reported by Tocco et al. in 45 patients with late SCFs [7]. In the study, these patients were selected after imagistic studies of the mediastinum, mainly CT scans, that ruled out deep infection. Also, patients in this group had serious comorbidities like severe renal insufficiency, cardiomyopathies, and chronic obstructive lung disease, pathologies that made surgical intervention difficult. Furthermore, more than half of the patients had previous failed treatment of SCFs, including steel wire removal of bone curettage. Antimicrobial therapy was prolonged from 3 months up to 30 months (mean 74 months) in patients with normal renal function. Two antibiotics were used, one according to the identified organism, and rifampicin as the second most commonly associated. In patients with negative wound cultures, empirical therapy against *Staphylococcus* was administered, and no glycopeptides were used for methicillin-resistant *Staphylococcus*, only teicoplanin. For CNS, fucidic acid and trimethoprim/sulphamethoxazole had great results, with caution regarding renal function and gastrointestinal complications. Only 5 relapses were recorded and were attributed to short-term antibiotic treatment. However, it should be mentioned that patients in this group were highly selected, those with no response to antibiotic therapy, those with multiple fistulas, and those with easy bleeding tissue protruding from the fistula being referred to additional surgical interventions.

Conservative surgery and antimicrobial therapy are other strategies used in patients with late SCFs. Steel wire removal and/or removal of the infected sternal bone and or costal cartilage are the most used techniques reported [21,24,25,26]. In cases where a large area of the sternum is removed, traditionally, a soft tissue flap is used to fill the remaining defect. The great omentum flap, the pectoralis muscle, or the rectus abdominus muscle flaps can be used [27,28,29]. However, complications like sternal instability with or without chronic pain are reported in 40% of cases, and even abdominal hernias after omental affect the quality of life of these patients. Tobakovi et al. report an extracellular matrix patch as an alternative for muscle flaps, a technique also described by Boulemden in a pediatric patient [2,30].

Modern techniques for sternal reconstruction and chest stability have emerged after large sternectomy. Chest stability can be achieved using various prosthetic materials such as two layers of methyl methacrylate mesh or a polytetrafluoroethylene patch coupled with a muscle flap for the soft tissue cover [31]. In cases with extensive resection that involve multiple ribs, titanium systems are used for reconstruction [32]. The titanium bars have an adaptable length and are anchored to the ribs to prevent fracture or dislocation, and besides maintaining chest stability, they prevent respiratory complications [33]. Also, modern technologies like electron-beam melting technology generate 3D-printed personalized stent prosthesis. Besides titanium, carbon fiber molds or alumina-ceramic models can be used for sternum implants [34]. Other strategies reported for sternal reconstruction include cryopreserved allografts and homografts from cadaveric donors [35], and regenerative approaches that promote bone regeneration using cell therapy based on mesenchymal stem cells [36].

In order to aid in the surgical management, Herrera et al. propose staining the fistula with methylene blue (fistulography) to determine the macroscopic extent during surgery [37]. However, imagistic diagnosis is essential in SCF’s management. A chest scan is most frequently used, but conventional images could be normal, especially if the SCF occurs under 21 days (30). Multidetector Computed Tomography has the advantage of superior bone resolution and has a 93.5% sensitivity and 96% specificity in detecting osteomyelitis [38]. Specific findings include cortical destruction, bony sequestrum or involucrum, sinus tract formation, demineralization, abscesses, and adjacent myositis when contrast is used. A dual energy chest scan that excludes calcium through the use of water density image can detect bone marrow edema, the earliest sign of osteomyelitis. Also, the use of high voltages is used, artifacts from the sternal wires can be eliminated [39]. MRI has superior sensitivity and specificity in the detection of sternal osteomyelitis and can detect bone marrow edema [39]. This early finding is reported as confluent hypointensity on T1-weighted images and hyperintensity on T2-weighted images, while loss of the normally hypointense cortex on T1- and T2-weighted images represents cortical destruction [39]. Scintigraphy with 99mTc- or WBC scintigraphy can be an alternative for MRI. This test is valuable, especially if the bone is normal, because high uptake can also be seen in fractures or degenerative processes [40].

Polymethylmethacrylate (PMMA), commonly known as acrylic bone cement, is a non-resorbable biomaterial that has been employed in orthopedic clinical practice for many decades. In prosthetic joint infections or chronic osteomyelitides, PMMA mixed with different antibiotics is used as a local antibiotic carrier in the first step of management, together with hardware removal and debridement [12]. PMMA is produced by combining a polymeric powder with a liquid monomer, triggering an exothermic polymerization reaction that yields a solid, rigid structure. To incorporate antibiotics or other agents, these compounds are blended into the polymer powder prior to monomer addition [12]. The elution of antibiotics from PMMA occurs through reciprocal diffusion and can be described in two sequential phases. The burst release takes place within minutes to hours, and produces high local concentrations of the antimicrobial agent. This rapid release is primarily a surface-driven event, in which antibiotics present on or near the surface of the cement dissolve into the surrounding body fluids [12]. The second stage, termed sustained release, begins after several days and maintains a lower but prolonged local antibiotic concentration. In this phase, water-soluble antibiotics gradually diffuse from within the cement matrix following penetration of water. Owing to the hydrophilic nature of PMMA, water molecules are attracted into the material, facilitating the slow liberation of these antibiotics into the surrounding tissues [41]. In the first hours to days after implantation, antibiotic concentrations released from the PMMA exceed the minimal inhibitory concentrations (MIC) of most pathogens [42]. Data regarding the prolonged antibiotic release concentrations are contradictory. While some data suggest that the PMMA antibiotic release concentrations are above MIC for several months [43,44], other authors report concentrations below MIC after one to two months [45]. High concentrations of antibiotics, especially gentamicin and clindamycin, are reported to reduce biofilm formation and bacterial adhesion, but not fully inhibit these processes [46]. This finding is important because biofilm decreases susceptibility to local and systemic antibiotics. The biofilm also acts as a barrier to antibiotics, decreasing transport rates via interactions with substances in the matrix, and also, reduced metabolism of bacteria inside the biofilm causes reduced cell growth and division, making them less sensitive to antibiotics [47]. High local concentrations increase the possibility of cytotoxicity and systemic toxicity. However, for most antibiotics used, the toxic concentrations are above local concentrations reported in vivo. Regarding systemic toxicity, multiple studies report serum concentrations below toxic thresholds when using PMMA with antibiotics; however, a meta-analysis reports cases of nephrotoxicity and acute renal failure [48,49,50].

The success rate for treatment of chronic osteomyelitis reported by Klemm et al., the group that first used antibiotic-loaded PMMA beads, was 91.4% [8]. Data from literature suggest an 80–100% chronic osteomyelitis eradication using this strategy [9,10,11,12,51]. It should be mentioned that none of the above studies did not analyzed osteomyelitis of the sternum after cardiac surgery. Table 1 summarizes the main studies in the field of cardiovascular surgery that use PMMA for the treatment of infection. To our knowledge, only one study reports this strategy after cardiac surgery in two patients in adults. Both patients had early deep sternal wound infection, approximately one week after discharge. Sternal heeling was achieved in both cases using gentamicin-impregnated polymethylmethacrylate chains [12].

### 3.4. Future Directions

Our findings highlight the importance of differentiating between localized and systemic infections after cardiac surgery. In the present case, the absence of systemic inflammatory response (normal CRP, WBC, and procalcitonin levels) strongly suggests that the infection was confined to the sternal site without significant dissemination. This underscores the clinical relevance of tailoring diagnostic and therapeutic strategies to the extent of the infectious process.

Emerging biomarkers such as serum butyrylcholinesterase (BChE) represent a promising avenue for improving early risk stratification and individualized management of surgical site infections [52]. Unlike traditional markers, BChE reflects both systemic inflammation and the patient’s metabolic and nutritional status. Decreased perioperative BChE levels have been correlated with higher SSI incidence, increased sepsis risk, prolonged intensive care stays, and higher postoperative morbidity. This dual role makes BChE a valuable adjunct to conventional inflammatory markers such as CRP and procalcitonin.

**Table 1:** Studies describing the use of PMMA.

Author	Year	Type	Cause of Infection	Outcome
Coghill et al. [53]	2018	Case report (3 year male)	Mediastinitis after cardiac surgery	Successful treatment
Genovese et al. [54]	2016	Retrospective review (6 adult patients)	prosthetic vascular graft infections	Successful treatment at Mean follow-up 7.3 ± 8.3 months
Chiu et al. [12]	2006	Case report (2 adult patients)	Mediastinitis	Successful treatment
Stone et al. [55]	2012	Retrospective review (42 adult patients)	lower extremity vascular surgical site infections	At the median follow-up of 17 months, the limb loss was 21.4% and the recurrent infection rate was 19.4% (seven of 36)

Future research should focus on establishing perioperative cut-off values for BChE that predict SSI severity, validating its diagnostic performance across diverse cardiac surgery populations, and integrating it into multimodal monitoring protocols. Prospective studies combining BChE with other novel biomarkers—such as interleukin panels, circulating cell-free DNA, or metabolomic signatures—could refine patient risk stratification and support real-time clinical decision-making. Additionally, longitudinal monitoring of BChE from the preoperative through the postoperative period may clarify its kinetics, improve early detection of systemic inflammation, and guide the escalation or de-escalation of antimicrobial therapy.

By embracing these future directions, clinicians may be able to shift from a reactive to a proactive approach in identifying and managing SSIs, ultimately improving outcomes, shortening hospital stays, and reducing the economic burden associated with postoperative infections in cardiac surgery.

## 4. Conclusions

This case highlights that late sternocutaneous fistula following cardiac surgery, although rare, requires a high index of suspicion and comprehensive imaging to assess the extent of underlying chronic sternal osteomyelitis. The use of antibiotic-loaded PMMA beads, in conjunction with thorough surgical debridement and targeted antimicrobial therapy, can provide an effective local drug delivery system and promote wound healing while preserving chest wall stability. Given that this therapeutic approach has been reported in only two other cases in the literature, our experience adds to the limited evidence supporting its use in post-sternotomy infections. Although our 6-month follow-up revealed no recurrence, longer and larger follow-up studies are warranted to confirm the durability of infection control in chronic osteomyelitis and biofilm-related settings.

## Data Availability

No new data were created or analyzed in this study.
